# Spontaneous celiacomesenteric trunk dissection: Case report

**DOI:** 10.1016/j.ijscr.2020.04.103

**Published:** 2020-05-21

**Authors:** Mabrouka Boukoucha, Abdelwahed Yahmadi, Hakim Znaidi, Raoudha Ben Khelifa, Alifa Daghfous

**Affiliations:** a Department of Radiology, Trauma and BurnCenter of Ben Arous, Tunisia; b Department of Surgery, Trauma and Burn Center of Ben Arous, Tunisia

**Keywords:** Celiacomesenteric trunk, Dissection, Mesenteric ischaemia, Acute abdominal

## Abstract

**Introduction:**

The celiacomesenteric trunk (CMT) is one of the most striking among the different variations of the normal vascularisation of the gastro-intestinal tract. It is often accidentally discovered during autoptical dissections, angiography or abdominal computed tomography (CT).

**Case presentation:**

A 27-year-old man was admitted to emergency for an acute abdominal pain. For his critical condition, the patient was immediately brought to the operating room. A extensive intestinal necrosis was found. Post-operator CT discovered a common CMT that is complicated by extended thrombosis. Despite all resuscitation measures, the patient died of septic shock two days later. An autopsy was performed showed that the main cause of intestinal ischemia was related to dissection of a common CMT.

**Discussion:**

A CMT is a highly unusual variation in humans. It is usually asymptomatic and may be discovered incidentally during vascular surgery, radiologic imaging, or cadaver's dissection. Lesion of this entity can lead to serious gastrointestinal complications including necrosis. Different classifications are proposed in the literature.

**Conclusion:**

As it is associated with the risk of mesenteric ischaemia, CMT should be kept in mind as a differential diagnosis for cases of recurrent non-specific abdominal pain.

## Highlights

• As it is associated with the risk of mesenteric ischaemia or necrosis of the gastro-intestinal tract, the commun celiacomesenteric trunk should be kept in mind as a differential diagnosis for cases of recurrent non-specific abdominal pain.
• When this vascular variant is observed, it is desirable; that a surgical treatment be planted to avoid any possible abdominal complications.

## 1 Introduction

The Celiacomesenteric trunk (CMT) is a very rare anatomical variant that is characterized by a common origin of the celiac trunk and superior mesenteric artery in from the aorta. It is an extremely rare occurrence, accounts for less than 1% of all celiomesenteric anomalies [1]. Recent imaging technics, particularly the computed tomography (CT) play a fundamental role in the diagnosis of this entity.

Injury of CMT can lead to serious gastrointestinal complications including bowel necrosis. In this document, we describe a case of CMT dissection leading to mesenteric ischaemia. To the best of our knowledge, this is the first case to be reported in the literature.

The purpose of this article is to review the appearance of this extremely rare entity, to evaluate the diagnostic utility of CT in the detection of this disease and to discuss the management methods. This work has been reported in line with the SCARE criteria [2].

## 2 Presentation of case

A 27-year-old man was admitted to emergency of a small peripheral hospital for an acute abdominal pain. Previously, he was healthy and had no remarkable antecedents, other than history of frequently smoking. According to his family, this abdominal pain had occurred several days before admission, and became so severe that he consulted for further examination and treatment.

In the emergency room, the patient was in a state of shock. His blood pressure was 80/60 mmHg and the pulse rate was 120 beats per minute. The physical examination was remarkable for abdominal distention with peritoneal signs. The results of laboratory data indicated that everything was within reference limits. The abdominal radiogram showed a nonspecific gas pattern.

With this critical condition patient, transfer to specialized surgical hospital was impossible and surgeons decided to operate. In operating room, a necrotic bowel extending from the upper ileum to distal ileum was found. A prolonged resection with an ileostomy was performed. However, no obvious etiology has been found to explain this ischemia.

Later, a CT scan was realized. So, a very rare anatomic variant was revealed. The coeliac trunk and the superior mesenteric artery arose as common trunk from the ventral surface of the abdominal aorta.

**Fig. 1:**
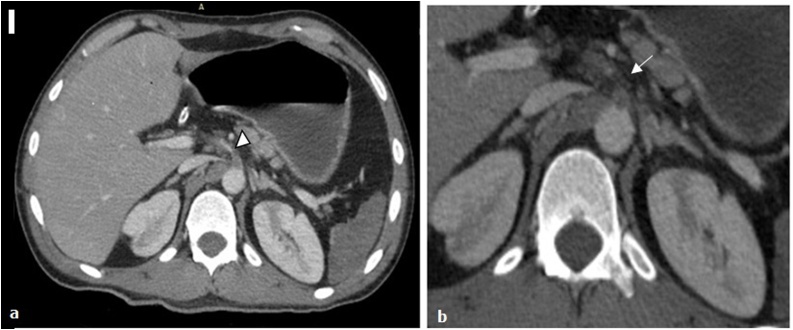
**a:** Axial CT showed an extended thrombosis (Arrowhead) of common celiacomesenteric trunk, the aorta is normal. **b**: A non-specific periarterial fat infiltration (Arrow) around this trunk was found.

Furthermore, an extended thrombosis of the common trunk to the superior mesenteric artery was diagnosed. This thrombosis was associated with a non-specific periarterial fat infiltration around (Fig. 1a, b). However, no intimal flap was detected no distal run off of the superior mesenteric artery was found. The inferior mesenteric artery was permeable with normal aortic origin.

**Fig. 2:**
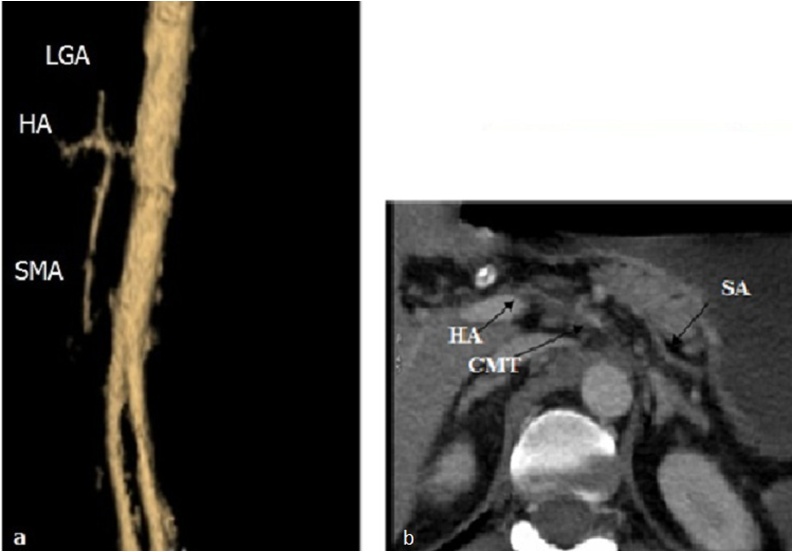
**a**: 3D volume rendered images showed a celiacomesenteric trunk divided in two branches: superior branche (hepato-gastro-splenic trunk or celiac trunk) and inferior branche superior mesenteric artery (SMA). **b**: This coeliac trunk gives his three classical branches (**b**): hepatic artery (HA) left gastric artery (LGA) and splenic artery (SA).

3D volume rendered images showed that this common trunk is at the level of the L1 vertebrae. It has a length of 10 mm before his bifurcation to a superior mesenteric artery and coeliac trunk which gives three classical branches; hepatic, splenic and left gastric arteries (Fig. 2a, b). The diagnosis of an extended CMT thrombosis to the superior mesenteric artery was retained.

An urgent administration of an anticoagulant was started and the patient was immediately transferred to another hospital to see the possibility of vascular management. Later, the patient was evolved with a septic state requiring his hospitalization in the reanimation unit. Despite all resuscitation measures, the patient died of septic shock two days later. For the goal of scientific interest, an autopsy was performed. Results showed that the main cause of extensive intestinal ischemia was related to thrombosed dissection of a common CMT. Unfortunately, the cause of dissection remained unknown, as no histological examination was realized. However, the interrogatory to his family revealed the history of Wegner granulomatosis in his sister.

## 3 Discussion

A CMT is a highly unusual variation in humans. It is usually asymptomatic and may be discovered incidentally during vascular surgery, radiologic imaging, or cadaver’s dissection [1]. However, in other mammalian species, the morphological appearance of a CMT or celiac trunk-bimesenteric is a common feature [3].

This vascular variant resulting from anomalies of embryonic development that is the failure of disruption, at an early stage of fetal development of the ventral longitudinal anastomosis between the third and fourth roots is arising from the primitive dorsal abdominal aorta [3]. Different classifications are proposed in the literature. The most widely used among them is that of Morita [4] who suggested 5 types of CMT variants. Our case belongs to type I’ of Morita’s classification, when the common trunk is divided in two branches, superior (hepato-gastro-splenic trunk or celiac trunk) and inferior (superior mesenteric artery).

Disease involving this rarely vascular anomaly is extremely uncommon. Only nine cases of aneurysmal dilatation and one case of thrombosis have been reported in the literature [5]. To our knowledge, no case of dissection has been previously reported. In our observation, despite no main cause of this CMT dissection was confirmed, many risk factors in relationship with of splanchnic arteries dissection were found, such as smoking and the history of granulomatosis in the family. Thus the dissection is probably secondary to an underlying vasculitis.

Multi slice tomography with arterial and portal phase acquisitions plays a major role to detect these diseases. It allows noninvasive assessment of normal anatomy and anatomic variant of celiac trunk. Three-dimensional images in combination with multiplanar reformation (MPR) images are used to study his anatomical origins and the major branches. In fact, in this present case, dissection was confirmed by autopsy and it was be not possible to view the intimal flap in CT. Based on the classification of superior mesenteric artery dissection established by Zerbib et al. [6], the dissection morphology of our CMT was typed VI with complete occlusion of true lumen. Yet, an increased diameter of the trunk with increased attenuation of the surrounding fat was found, as like in all cases of a dissection of the visceral arteries noted in literature [7]. That is why; dissection must be included in the differential diagnosis in cases of luminal thrombosis in an enlarged vessel with no definite intimal flap.

Theoretically, if interruption of circulation in superior mesenteric artery, the residual lumen, the collateral circulation from celiac trunk and the inferior mesenteric artery should provide proper blood flow to avoid ischemia. Thus, the thrombosis of the origin of CMT had a lethal effect because it caused the full stoppage of the splanchnic arterial supply and can lead to the necrosis of the gastro-intestinal tract [5]. So, CMT should be kept in mind as a differential diagnosis for cases of recurrent non-specific abdominal pain and once it is observed, it is desirable; that a surgical treatment be planifed to avoid any possible abdominal complications. Our case may provide useful information for clinical applications in surgeries of the abdomen. The knowledge of variations concerning the celiac trunk and the superior mesenteric artery are of extreme clinical importance in the areas of surgical and interventional procedures.

Given that the dissection of common CMT is extremely rare, optimal management has not yet been established and in the both cases of CMT thrombosis previously reported [5,8], surgical intervention failed to rescue patients.

In literature, the choice of the repair method of splanchnic arteries dissection has been applied depending on the severity of the dissection and the patient’s hemodynamic status. In the absence of visceral mal perfusion and as long as the patency of the vessel is well compensated by the collateral circulation, most authors recommend initial conservative treatment [9]. But, in the situation of CMT dissection, this solution seems to be impossible, given the occlusion of the vessel would be not well compensated by the collateral circulation. However, when the patient is hemodynamically unstable, two endovascular approaches have been proposed as the preferential method, the coil embolisation and the stent-graft. As if the experience with the coil embolisation schowed that there is a potential risk of ischemic complications due to the difficulty in preserving flow through the branches of celiac artery, the stent-graft was suggested as a valid alternative endovascular treatment, which better preserves the distal flow blood [10]. In fact, only the endovascular stent placement in spontaneous superior mesenteric artery dissection was reported [11].

Nowadays, open surgery is only recommended when bowel necrosis or peritoneal irritation is found and when endovascular treatment fail. So, for emergency, it may be necessary that physicians performing endovascular treatment cooperate with ones doing gastrointestinal surgery.

## 4 Conclusion

The CMT is an exceptionally rare anatomical variation and when it is observed, it is desirable; that a surgical treatment be planifed to avoid any possible abdominal complications. This can be easily achieved by multidetector computed angiography.

## Declaration of Competing Interest

We have no conflicts of interest to disclose.

## Funding

We have no source of funding for this reported case.

## Ethical approval

The case is exempt from ethical or ethnical approval, according to Tunisian Medical Ethics Committee.

## Consent

“Written informed consent was obtained from the patient’s guardian for publication of this case report and accompanying images. A copy of the written consent is available for review by the Editor-in-Chief of this journal on request”.

## Author contribution

Study conception: Mabrouka Boukoucha.

Data collection: Alifa Daghfous, Abdelwahed Yahmadi, Hakim Znaidi.

Writing: Mabrouka Boukoucha.

Critical review and revision: all authors.

Final approval of the article: all authors.

## Registration of research studies

This case is not interested to be registered in a publicly accessible database.

## Guarantor

Mabrouka Boukoucha.

## Provenance and peer review

Not commissioned, externally peer-reviewed.
